# Virtual reality in ophthalmology education: simulating pupil examination

**DOI:** 10.1038/s41433-022-02078-3

**Published:** 2022-05-10

**Authors:** Michael Williams

**Affiliations:** School of Medicine, Dentistry and Biomedical Science, Queen’s University of Belfast, Belfast, UK

**Keywords:** Health services, Education

Scantling-Birch et al. [1] describe technology enhanced learning (TEL), and the advantages of TEL. We describe a virtual reality (VR) model for pupillary examination.

Passive learning, from textbooks or online content, has some value, but retention is better with interaction, reflection and also through experiential learning: doing. Pupillary examination is obviously central to ophthalmic assessment, but also highly relevant for those managing a range of emergency, neurological and general medical presentations. Medical students can practice pupillary examinations on each other, though will have normal reactions. Simulated patients can be pharmacologically dilated, but they are a limited resource, and no other abnormality can be simulated. The challenge is for students to have seen a range of pupil abnormalities before they’re in a cubicle with a real patient. So, they are sent to as many clinical settings as possible, sometimes with a logbook, in the hope that they all get enough opportunities to see, and maybe even to examine abnormal pupils. It works generally, but for seeing a range of pupil abnormalities, its opportunistic. One solution seems obvious: virtual reality.

I have worked with a VR coding company to develop an application for pupillary examination (Sentireal: https://www.sentireal.com/post/virtual-clinical-classroom-ophthalmology-student-vr-training Accessed March 2022). The app is developed for the Oculus headsets. The user starts in a clinic reception room (Fig. 1), where they select one of 12 different options: 10 different pupil abnormalities (like right APD, left fixed dilated pupil with ptosis, right fixed dilated pupil with red eye etc.), random and normal options. Then they enter the clinical room, where the patient awaits with whatever pupil abnormality has been selected. The user’s point of view can be cast to any device on the same wifi network. The user picks up a pen torch, turns it on and examines the pupils, using the same physical movements as would be used in real life (Fig. 2), swinging the torch between eyes, or moving towards the patient for a closer look for example.

**Fig. 1:**
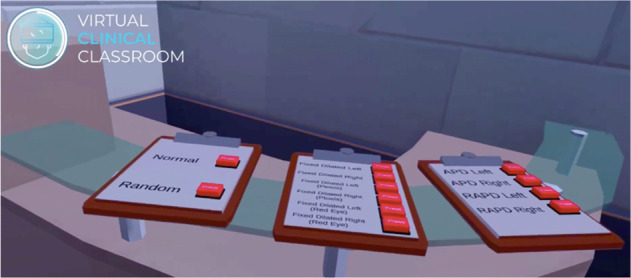
A user’s view in the clinic reception room. Users have to move their hands to press one of the buttons to choose the pupil abnormality.

**Fig. 2:**
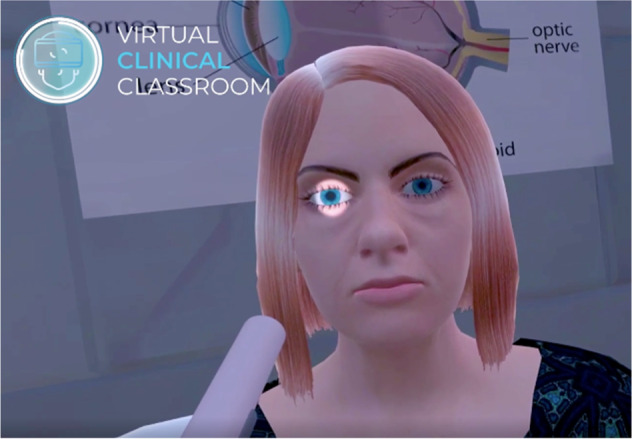
A user’s view while examining pupils. The pen torch is held and moved as in real life.

It has been used with over 250 final year medical students in Queen’s University of Belfast, as part of a session called ‘The Simulated Eye Clinic’. Most had never used VR before, and there was usually a shocked but delighted reaction when they put the headset on and immediately became immersed into a virtual world. I learnt how to instruct students and get them examining the pupils within 30–60 s. For example, I learnt seemingly obvious things like showing them the only two buttons they’d need on the controller before they put the headset on, and going in myself first to choose the pupil abnormality, so the student started in front of the patient. Students worked in pairs, so the 1st student to do it then taught the 2nd. With ten minutes per pair, over 250 students got to examine an afferent pupillary defect, and a fixed dilated pupil in a red eye, i.e. acute glaucoma. When surveyed, 96% of 69 respondents, asked opportunistically in batches, said even this short experience improved their knowledge and confidence of examining pupils. Comments included:

“…allowed me to see an APD which I’d never seen before!”

“I LOVED the VR – something so innovative and a bit different to keep the learning process interesting!!”

“…takes the anxiety out of performing in front of students + so you can make mistakes”

VR is merely a tool, and rather than being enticed by its novelty, it must be used as part of an overall educational strategy, to deliver specific learning outcomes. It has potential use for assessment too.

Deliberate practice is used to learn skills, and depends on repitition, intentionality, faith and fun (‘RIFF’). In a cost-effective way, VR provides an opportunity for students to engage in clinically relevant deliberate practice, with an SP who will never tire, and have whatever abnormality we choose her to have. Never to be a replacement for learning with real patients, it is a tool to accelerate preparation for meeting real patients.

## Data Availability

Original data are in the possession of the author and are available on request.

## References

[1] Scantling-Birch Y, Naveed H, Tollemache N, Gounder P, Rajak S (2022). Is undergraduate ophthalmology teaching in the United Kingdom still fit for purpose?. Eye.

